# Discovery of Antimicrobial Peptides That Can Accelerate Culture Diagnostics of Slow-Growing Mycobacteria Including *Mycobacterium tuberculosis*

**DOI:** 10.3390/microorganisms11092225

**Published:** 2023-09-02

**Authors:** Kai Hilpert, Tulika Munshi, Paula M. López-Pérez, Joana Sequeira-Garcia, Sven Hofmann, Tim J. Bull

**Affiliations:** 1 Institute of Infection and Immunity, St George’s, University of London, Cranmer Terrace, London SW17 0RE, UKtbull@sgul.ac.uk (T.J.B.); 2 TiKa Diagnostics Ltd., Cranmer Terrace, London SW17 0RE, UK

**Keywords:** antimicrobial peptides, MTB, tuberculosis, mycobacteria, TB diagnostics, MAP, Johne’s disease, bacterial growth, culture, *Mycobacterium tuberculosis*

## Abstract

Antimicrobial peptides (AMPs) can directly kill Gram-positive bacteria, Gram-negative bacteria, mycobacteria, fungi, enveloped viruses, and parasites. At sublethal concentrations, some AMPs and also conventional antibiotics can stimulate bacterial response increasing their resilience, also called the hormetic response. This includes stimulation of growth, mobility, and biofilm production. Here, we describe the discovery of AMPs that stimulate the growth of certain mycobacteria. Peptide 14 showed a growth stimulating effect on *Mycobacteria tuberculosis* (MTB), *M. bovis*, *M. avium* subsp. *paratuberculosis* (MAP), *M. marinum*, *M. avium-intracellulare, M. celatum,* and *M. abscessus.* The effect was more pronounced at low bacterial inocula. The peptides induce a faster transition from the lag phase to the log phase and keep the bacteria longer in the log phase before entering stationary phase when compared to nontreated controls. In some cases, an increase in the division rate was observed. An initial screen using MAP and a collection of 75 peptides revealed 13 peptides with a hormetic effect. For MTB, a collection of 25 artificial peptides were screened and 13 were found to reduce the time to positivity (TTP) by at least 5%, improving growth. A screen of 43 naturally occurring peptides, 11 fragments of naturally occurring peptides and 5 designed peptides, all taken from the database APD3, identified a further 44 peptides that also lowered TTP by at least 5%. Lasioglossin LL-III (Bee) and Ranacyclin E (Frog) were the most active natural peptides, and the human cathelicidin LL37 fragment GF-17 and a porcine cathelicidin protegrin-1 fragment were the most active fragments of naturally occurring peptides. Peptide 14 showed growth-stimulating activity between 10 ng/mL and 10 µg/mL, whereas the stability-optimised Peptide 14D had a narrow activity range of 0.1–1 µg/mL. Peptides identified in this study are currently in commercial use to improve recovery and culture for the diagnostics of mycobacteria in humans and animals.

## 1. Introduction

*Mycobacterium tuberculosis* (MTB) is the primary causal agent of tuberculosis in humans. MTB belongs to the *M. tuberculosis* complex (MTBC) which includes the closely related species *M. bovis*, *M. africanum*, *M. microti*, *M. pinnipedii*, *M. caprae*, and *M. canetti*. The slow-growing *M. tuberculosis* divides in conventional culture media every 16–20 h [1]. According to the Centres for Disease Control and Prevention (CDC), one-fourth of the world’s population is infected with tuberculosis (TB) [2]. The World Health Organization (WHO) estimated 10.6 million TB cases in 2021, causing 1.6 million TB-related deaths worldwide [3].TB is a leading killer among human immunodeficiency virus (HIV)-infected people with weakened immune systems. In recent years, the treatment of TB has become more challenging due to the emergence of multi- and extreme drug-resistant strains (MDR- and XDR-TB).

*Mycobacterium avium* subsp. *paratuberculosis* (MAP) causes Johne’s disease, a chronic enteritis that can lead to severe economic losses in sheep, goat, and dairy cattle farms. The disease has also been reported in primates, horses, pigs, deer, alpacas, llamas, rabbits, stoats, foxes, and weasels [4]. The huge excretion of MAP from clinically diseased animals contaminates milk and meat supplies and is detected in pasteurised milk (including infant milk formula) and pastureland wash-offs which carry over through treatment plants to remain viable in potable water supplies. MAP is also a slow-growing organism, often taking months to generate visible colonies.

Antimicrobial peptides (AMPs), also called host defence peptides, represent a ubiquitous response in nature to overcome microbial infections or to compete for an ecological niche [5]. The antimicrobial activities can include actions against Gram-negative and Gram-positive bacteria, including mycobacteria, fungi, and enveloped viruses [6,7,8,9,10]. Of particular interest is their ability to kill multidrug-resistant bacteria [11]. Within the last two decades, it has become increasingly clear that various antimicrobial peptides play a role in regulating the process of innate immunity. It has been reported that some AMPs can have direct and indirect chemotactic functions, regulate chemokine and cytokine production, and promote wound healing [12,13,14,15,16,17]. With the multitude of cationic peptide sources, structures, and spectra of activity come a number of complex and controversial structure-function theories attempting to describe and explain peptide modes of interaction that kill bacteria [18]. Most AMPs bind avidly to the lipopolysaccharides (LPSs) of Gram-negative bacteria and lipoteichoic acids of Gram-positives, and subsequently associate with and depolarise and/or permeabilize cytoplasmic membranes [19]. In many cases, the peptide translocates into the microbial cell and attacks internal targets. AMPs can therefore affect very different processes, and most likely have often not one target, but multiple. It has been reported that some AMPs bind to lipid II, DNA and RNA, chaperons and ribosome, proteases, adenosine triphosphate (ATP), and ABC transporter [18,20,21,22,23,24,25,26,27,28]. At sublethal concentrations, some AMPs and antibiotics, in general, can stimulate growth, mobility, frequency of mutation, and plasmid conjugative transfer [29]. For example, GL13K peptides have shown growth stimulation and an increase in metabolic activity for *Pseudomonas aeruginosa* [30]. This effect is called a hormetic response.

WHO estimates that about 60 million tests for human TB are carried out per year. The most common test is a microscopic evaluation of stained sputum smears which is simple, rapid, and inexpensive. However, not only is the sensitivity of this method compromised when the bacterial load is low (less than 1 × 10^4^ organisms/mL), but it also performs poorly in extrapulmonary tuberculosis, paediatric tuberculosis, and patients coinfected with HIV [31,32,33]. Other rapid tests like GeneXpert and interferon-gamma release assays (IGRAs) are now available but have their limitations. Limitations for DNA tests include susceptibility to inhibitors in some samples, and amplicon contamination leading to false positive reporting. Antibiograms through single-nucleotide polymorphisms (SNPs) can only detect known resistance mechanisms. IGRA does not differentiate between active and latent infection and is not useful in some children, immunocompromised patients, and during early acute infection. Culturing *M. tuberculosis* is the gold standard because it can identify and quantify bacterial load and allow antibiotic sensitivities or strain typing to be tested. However, worldwide, only 3–4 million samples are sent for culture per year. This is due to the need for infrastructure and training, cost, and the long-held perception that MTB grows too slowly to help initial diagnosis.

During a project to find antimicrobial peptides effective against *Mycobacterium avium* subsp. *paratuberculosis* (MAP), we unexpectedly came across a group of peptides that had a unique property of stimulating growth (referred to as hormetic response [29,30]). This study aimed to further investigate the characteristics of these peptides. We started by investigating whether this hormetic response could also be observed in other slow-growing mycobacteria, such as MTB. Surprisingly, we found that some peptides were capable of inducing the hormetic response, including MTB. This finding presents an exciting opportunity to develop peptides that could enhance routine culture-based diagnostics for MTB. Based on this discovery, we decided to screen more peptides against MTB and also assessed their ability to reduce time to positivity (TTP) in a routine diagnostic setting. For specific peptides, we explored dose dependency and measured the effects of more stable D-enantiomers. The peptides identified in this study have subsequently been used in a clinical trial (to be published elsewhere), demonstrating the superiority of diagnosing MTB using these peptides compared to a standard method.

## 2. Methods

### 2.1. Peptides

From the 139 peptides used in the study, 2 peptides were purchased at Bachem (Bubendorf, Switzerland), 35 at Umpep (Nordwestuckermark, Germany), and 40 at Kinexus Bioinformatics Corporation (Vancouver, BC, Canada). A total of 62 peptides were synthesized in our laboratory by automated solid-phase peptide synthesis (SPPS) on a MultiPep RSI peptide synthesizer (Intavis, Tuebingen, Germany) using the 9-fluorenyl-methoxycarbonyl-tert-butyl (Fmoc/tBu) strategy. Reactive side chains were protected by *t*Bu (Tyr and Asp), trityl (Trt, for Asn, Cys, Gln and His), 2,2,4,6,7 pentamethyldihydrobenzofuran-5-sulfonyl (Pbf, for Arg), and *tert*-butoxycarbonyl (Boc, for Lys and Trp). For automated SPPS, four equivalents of Fmoc amino acids (Bachem, Bubendorf, Switzerland) were coupled on TentaGel^®^ HL RAM resin (25 μmol scale, loading 0.3–0.4 mmol/g, Rapp Polymere, Tuebingen, Germany) after in situ activation with four equivalents of N,N,N′,N′-Tetramethyl-O-(1H-benzotriazol-1-yl)uronium hexafluorophosphate (HBTU; Carbosynth, Berkshire, UK) and eight equivalents of N-Methylmorpholine (NMM, Sigma, Dorset, UK). After a double-coupling procedure (2 × 30 min), the Fmoc group was cleaved using 20% (*v*/*v*) piperidine (Thermofisher Acros Organics, Geel, Belgium) in dimethylformamide (DMF, Jencons-VWR, Leicestershire, UK). Peptide amides were cleaved from the resin with 95% (*v*/*v*) aqueous trifluoroacetic acid solution (TFA, Fisher Scientific, Loughborough, UK) containing 5% (*v*/*v*) triisopropylsilane (TIPS, Thermofisher Acros Organics, Geel, Belgium)/water (1:1) scavenger mixture within 3 h. Cleaved peptides were precipitated from ice-cold methyl *tert*-butyl ether (MTBE; Thermofisher Acros Organics, Geel, Belgium). After washing and collection by centrifugation, crude peptides were dissolved in 20% (*v*/*v*) acetonitrile (ACN, Jencons-VWR, Leicestershire, UK)/80% (*v*/*v*) water containing 1% (*v*/*v*) TFA to a concentration of 15 mg/mL and analysed by analytical reversed-phase (RP) HPLC on a Shim-pack VP-ODS (120 Å, 150 × 4.6 mm, Shimadzu, Milton Keynes, UK) using a Shimadzu LC2010AHT system (Shimadzu, Milton Keynes, UK). The binary solvent system contained 0.1% (*v*/*v*) TFA in H_2_O (solvent A) and 0.1% (*v*/*v*) TFA in acetonitrile (solvent B). The identity was verified by a liquid chromatography–electrospray ionisation mass spectrometry (LC-ESI-MS) Shimadzu LC2020 system (Shimadzu, Milton Keynes, UK) equipped with a Jupiter 4 μ Proteo C18 column (90 Å, 250 × 4.6 mm, Phenomenex, Cheshire, UK). The binary solvent system contained 0.01% (*v*/*v*) TFA in H_2_O (solvent A) and 0.01% (*v*/*v*) TFA in acetonitrile (solvent B).

Selected crude peptides were purified to the homogeneity of >92% by preparative RP HPLC on a Shimadzu LC2020 system equipped with a Jupiter 10 μ Proteo C18 column (90 Å, 250 × 21.2 mm, Phenomenex, (Phenomenex, Cheshire, UK)) using a linear gradient system containing 0.01% (*v*/*v*) TFA in H_2_O (solvent A) and 0.01% (*v*/*v*) TFA in acetonitrile (solvent B). Pure products were finally characterized by analytical RP-HPLC and LCMS.

### 2.2. Mycobacterial Culture

The following strains were used: *Mycobacterium marinum* NCTC 2275, *Mycobacterium branderi* (clinical human isolate), *Mycobacterium intracellulare* serotype 7 NCTC10682, *Mycobacterium celatum* (clinical isolate), *Mycobacterium gastri* ATCC25158, *Mycobacterium abscessus* (clinical human isolate), *Mycobacterium porcinum* strain L53 (buffalo milk isolate), *Mycobacterium fortuitum* ATCC6841, *Mycobacterium vaccae* NCTC10916, *Mycobacterium smegmatis* (laboratory strain MC2155), *Mycobacterium avium* subspecies paratuberculosis (MAP, cattle isolate), and *Mycobacterium tuberculosis* (laboratory strain H37Rv). All mycobacterial species used in this study were grown in Middlebrook 7H9 media (BD, Wokingham, UK) with 0.2% glycerol (Sigma, UK), 0.1% casitone (Oxoid, UK), and 10% OADC (Becton Dickinson, Wokingham, UK) at 37 °C (except *M. marinum,* which was grown at 30 °C). For MAP, Mycobactin J (IDVet, Grabels, France) was added to the above growth media (final 2 µg/mL). Initial screening assays with peptides were carried out in 1.6 mL polystyrene semi-micro cuvettes (Fisherbrand, Loughboroughe, UK) with lids sealed with parafilm. Growth was measured by estimating optical density using a spectrophotometer every 2 days. Further confirmatory analysis was carried out in a BACTEC MGIT 320 automated mycobacterial detection system using 7 mL MGIT (Becton Dickinson, UK) mycobacterial culture tubes, supplemented with 0.8 mL oleic acid, albumin, dextrose, and catalase (OADC) and PANTA (antibiotic mixture) growth supplement prepared according to manufacturer recommendations.

### 2.3. Peptide Screening

Initial assays to screen for peptide activity on mycobacterial growth used 1 mL culture of Middlebrooks 7H9 (Difco, Oxford, UK) plus 10% OADC (Becton Dickinson, UK) and 2 µg/mL Mycobactin J (IDVet, Grabels, France) inoculated to 0.1 optical density at 600 nm (OD_600_) from exponential growth starter cultures in semi-micro cuvettes (Fisherbrand, Loughborough, UK) with lids sealed with parafilm. Growth (37 °C) was measured by OD_600_ every 2 days. Further confirmatory analysis was carried out in a BACTEC MGIT 320 automated mycobacterial detection system using 7 mL MGIT (Becton Dickinson, UK) mycobacterial culture tubes, supplemented with 0.8 mL OADC/PANTA growth supplement prepared according to manufacturer recommendations being inoculated as previously with exponential starter cultures and peptides up to various test final concentrations. Growth curves were derived by retrieving growth index values, based on the relative fluorescence of a proprietary oxygen depletion indicator, each day from an automated reader.

### 2.4. Quantitative PCR 

qPCR was performed using a CFX 96 machine (BioRad, Hercules, CA, USA) with each reaction containing 500 nM mycobacteria-specific Taqman primers cydA-F (5′ GACCACCAAGGCAAGCTGA 3′), cydA-R (5′ TCGCACAACGATTCGGC 3′) and 200 nM labelled probe cydA-P (5′ FAM-CATCTTCATCGGCTGCTGCTGGAA-TAMRA 3′) in 10 µL of 2x SsoAdvanced Universal Probes supermix (BioRad, USA) according to manufactures recommendations. Conditions used were 98 °C for 2 min; 58 °C for 30 s; 72 °C for 10 min. MTB H37Rv was grown as detailed above and incubated in the presence (1 µg/mL, 10 µg/mL, and 50 µg/mL) or absence of peptides. All samples were carried out in duplicates, incubated in the MGIT system and removed only when the control (without peptide) went positive. Sterile water was used as a negative control. The culture tubes were centrifuged at 10,000× *g* for 10 min and DNA was extracted using a Qiagen DNeasy blood and tissue kit (Qiagen, Manchester, UK). An MTB H37Rv gDNA dilution series (assuming 1 ng = 2 × 10^5^ genome equivalents) was used as a calibration curve in all qPCR reactions to estimate copy number for all experimental samples. 

## 3. Results

### 3.1. Initial Screen against Mycobacterium avium *subsp.* paratuberculosis (MAP)

**Figure 1:**
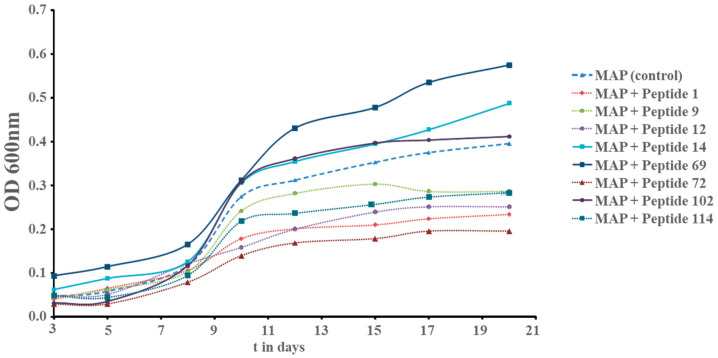
Growth curve of *Mycobacterium avium* subsp. *paratuberculosis* (MAP) with and without peptide treatment at 20 µg/mL. Growth was followed by measuring the optical density (OD) at 600 nm.

The first screen was originally designed to identify peptides that could inhibit the growth of *Mycobacterium avium* subsp. *paratuberculosis* (MAP) as a starting point for the development of an antimicrobial drug candidate. A collection of 75 peptides from various projects was created, that had already shown antimicrobial activity against other microbes [10,34,35,36]. The screen was performed at 20 µg/mL to find the most active inhibitory peptides. We expected to find two classes of peptides: (1) peptides that did not affect growth with a growth curve similar to the control and (2) peptides with antimicrobial activity with reduced growth resulting in a growth curve below the control. Of the 75 peptides, 41 peptides did not change the growth curve and 21 peptides showed growth inhibition. Unexpectedly, however, the results revealed a third class of peptides where the growth in the presence of peptides increased compared to the control, indicating a hormetic response. In total, 13 peptides were identified with a hormetic response, with peptides 14 and 69 showing the strongest effects (see Table S1A–C in the Supplementary Materials). Peptide 102, a version of Peptide 69 with a C-terminal modification, showed a slightly reduced effect compared to Peptide 69. The experiment was repeated with a smaller number of peptides to confirm this unexpected effect (see Figure 1).

**Figure 2:**
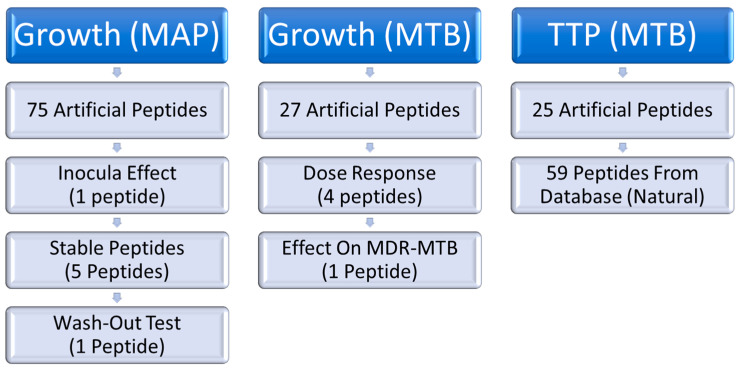
Schematic representation of experiments performed in this study. Growth means studies that follow the growth from start to end, whereas TTP (time to positivity) just looks at the early stages of growth until the mycobacteria can be detected by an MGIT in a BD BACTECMGIT 320 system. MAP stands for *Mycobacterium avium* subsp. *paratuberculosis*, MTB for *Mycobacterium tuberculosis,* and MDR for multidrug resistance. Stable peptides refer to peptides that are not easily degraded by proteases.

As a consequence of this discovery, a series of experiments was performed to study which peptides can induce growth stimulation and at what concentrations (see Figure 2).

**Figure 3:**
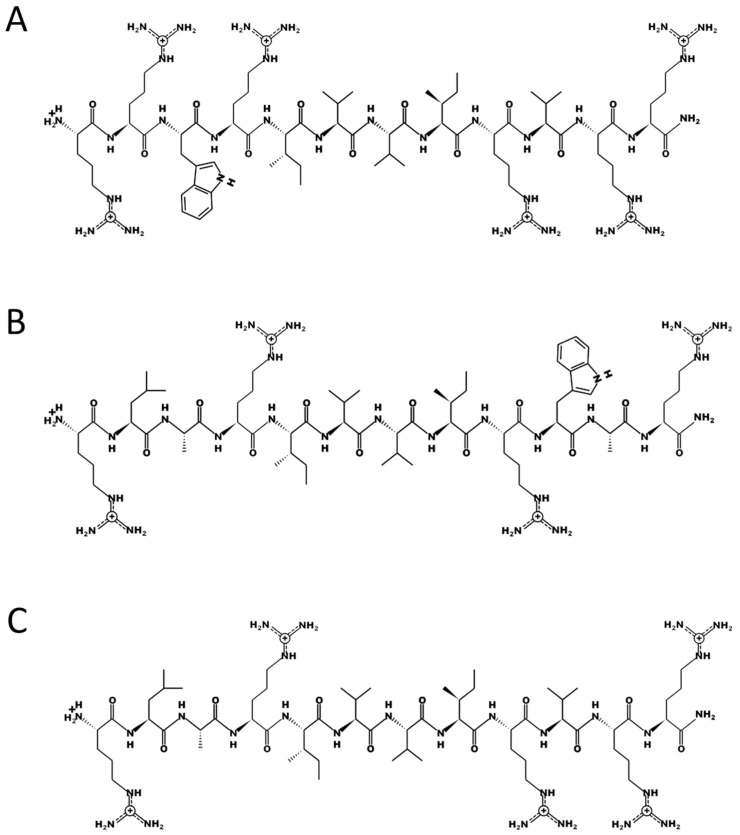
Primary structure of three 12mer antimicrobial peptides. (**A**) Peptide 69 (RRWRIVVIRVRR-CONH_2_), which improved growth, (**B**) Peptide 72 (RLARIVVIRWAR-CONH_2_), which reduced growth, and (**C**) Peptide 70 (RLARIVVIRVRR-CONH_2_), which showed no effect on growth. PEPDRAW was used to generate these structures (http://pepdraw.com/) (accessed on 14 September 2019).

These data showed that the inclusion of any cationic peptide was insufficient to cause an effect. There is a peptide-sequence-dependent activity occurring. A comparison of peptide sequences from each different activity class (reduced growth, normal growth, and accelerated growth) revealed that small changes in the primary structure can result in a strong effect on their biological activity. For example, Figure 3 illustrates three 12mer peptides containing the conserved core motif “RIVVIR”, which is also present in the bovine antimicrobial peptide bactenecin (RLCRIVVIRVCR-CONH_2_). Peptide 70 (RLARIVVIRVRR-CONH_2_) exhibited no discernible effect on the growth of *Mycobacterium avium* subsp. *paratuberculosis* (MAP). However, introducing minor alterations to the N-terminal region of the peptides, involving the substitution of only two amino acids, resulted in an increase in positive charge (R) and enhanced hydrophobicity, along with improved membrane integration conveyed by tryptophan (W). These modifications, in turn, led to a noticeable acceleration in the growth of MAP, as evidenced by the behaviour of Peptide 69 (RRWRIVVIRVRR-CONH_2_). On the other hand, by reducing the positive charge (R ->A) and enhancing hydrophobicity, along with an increased ability to interact with membranes (A, W) at the C-terminal region of the peptides, we observed a significant reduction in the growth of MAP for Peptide 72 (RLARIVVIRWAR-CONH_2_).

### 3.2. Test of Growth-Enhancing Activity in Other Mycobacterial Species and Inoculum Effect

**Figure 4:**
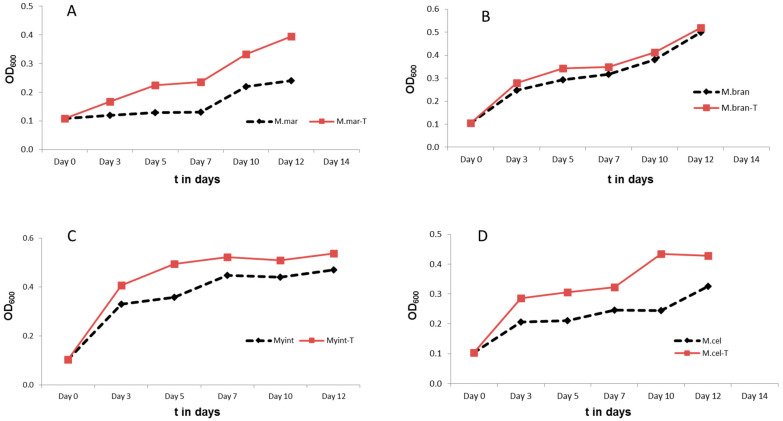

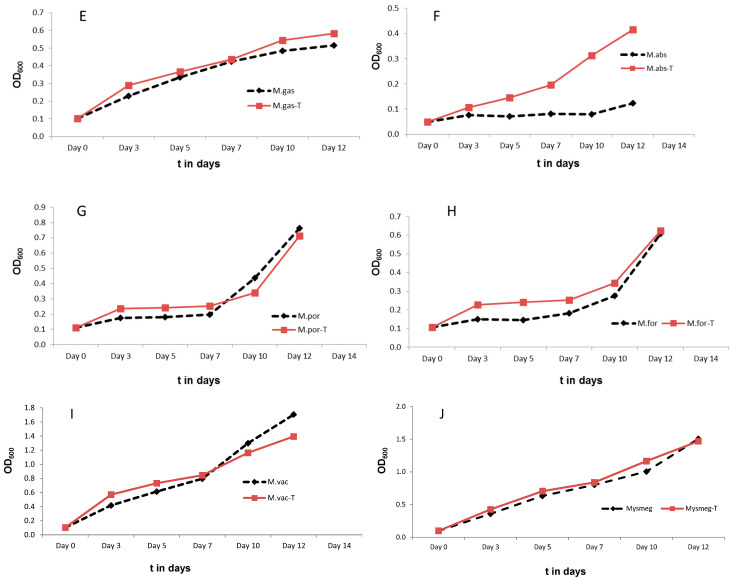
Monitoring the growth curve of slow- and fast-growing mycobacteria with and without Peptide 14 at 20 µg/mL. T in the legend indicates treatment with Peptide 14. The slow-growing mycobacteria included (**A**) *Mycobacterium marinum*, (**B**) *Mycobacterium branderi*, (**C**) *Mycobacterium avium-intracellulare* and (**D**) *Mycobacteria celatum.* The fast-growing mycobacteria included (**E**) *Mycobacterium gastri*, (**F**) *Mycobacterium abscessus*, (**G**) *Mycobacterium porcinum*, (**H**) *Mycobacterium fortuitum*, (**I**) *Mycobacterium vaccae,* and (**J**) *Mycobacterium smegmatis*.

Peptide 14 (WKIVFWWRR-CONH_2_) was selected and tested for growth-enhancing activity in other mycobacterial species, including slow-growing mycobacterial species *Mycobacterium marinum*, *Mycobacterium branderi*, *Mycobacterium avium-intracellulare*, and *Mycobacterium celatum*, and fast-growing mycobacterial species *Mycobacterium gastri*, *Mycobacterium abscessus*, *Mycobacterium porcinum*, *Mycobacterium fortuitum*, *Mycobacterium vaccae*, and *Mycobacterium smegmatis.* Peptide 14 was added to the cultures at 20 µg/mL and the OD_600_ was measured over time. The results in Figure 4 show that Peptide 14 was able to enhance the growth of most slow-growing mycobacteria (except *Mycobacterium branderi)* and one fast-growing mycobacterium (*Mycobacterium abscessus*).

**Figure 5:**
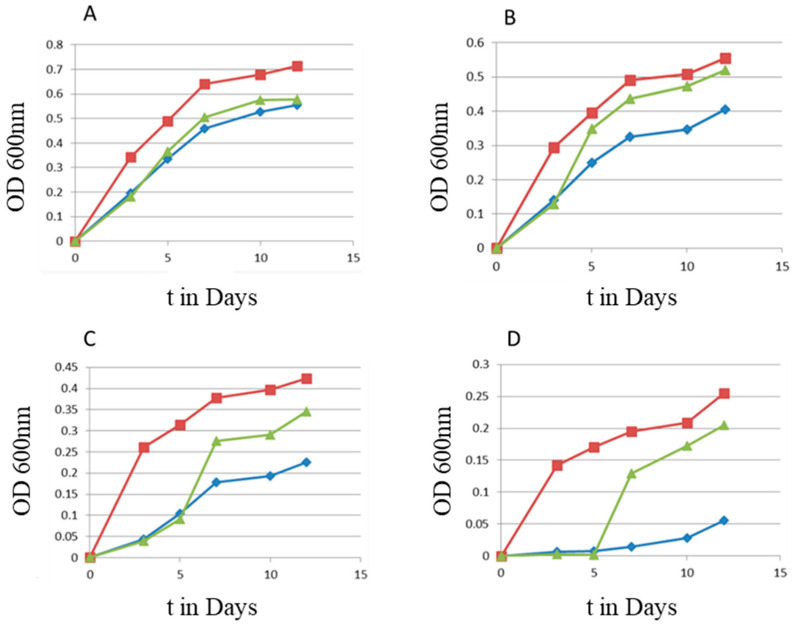
Growth of MAP with Peptide 14 at 20 µg/mL (red squares) and without peptide treatment (blue diamonds) monitored using the absorbance at 600 nm. Peptide 14 was added either directly to the diluted culture or on day 3 (green triangles) of the experiment. Different starting inoculum sizes were used, measured by absorbance at 600 nm: (**A**) 0.1, (**B**) 0.05, (**C**) 0.01, and (**D**) 0.001.

Peptide 14 was then tested for growth-enhancing effects in relation to the starting inoculum and time point of treatment. An exponentially growing culture of MAP was subcultured and regrowth was monitored. Peptide 14 was added at 20 µg/mL directly after the dilution or after 3 days (see Figure 5). The degree of effect from the peptide was dependent on the size of the starting inoculum, with the lower inocula being more greatly affected. Improved growth stimulation through the addition of peptide during the growth phase was also demonstrated (day 3).

### 3.3. Peptides Stabilized against Protease Cleavage

**Figure 6:**
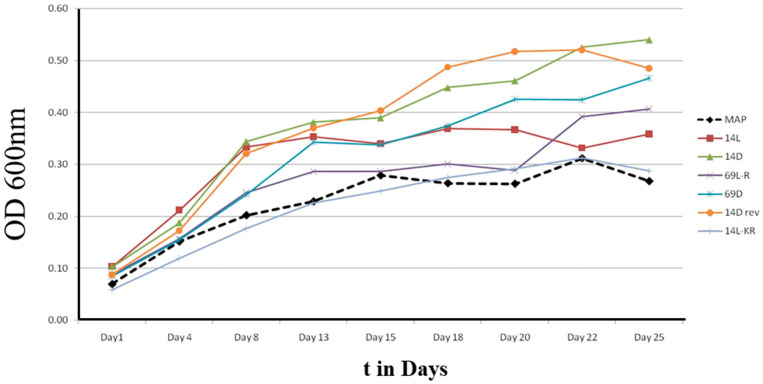
Growth of MAP with and without several peptides at 10 µg/mL measured by absorbance at 600 nm. 14L stands for Peptide 14 (WKIVFWWRR-CONH_2_), 14D for Peptide 14D (wkivfwwrr-CONH_2_), 14 rev for the retro-inverse sequence (rrwwfvikw-CONH_2_), 14 L-KR for an ornithine exchange peptide (WOIVFWWOO-CONH_2_), 69D for Peptide 69D (rrwrivvirvrr-CONH_2_), and 69L-R for an ornithine exchange variant (OOWOIVVIOVOO-CONH_2_).

During many days of mycobacterial culture, growth-enhancing peptides are exposed and possibly degraded by secreted and cell-surface-located proteases expressed by the bacteria themselves. We thus tested whether several modifications to increase the proteolytic stability of the peptides including a stereo-enantiomer of Peptide 14 showed any further growth effect. Peptide 14D (wkivfwwrr-CONH_2_), the retro-inverse sequence 14D-rev (rrwwfvikw-CONH_2_), and a version of Peptide 14, where proteolytic sensitive residues (arginine and lysine) were exchanged against ornithine (O) resulting in Peptide 14L-KR (WOIVFWWOO-CONH_2_), were synthesized [37]. In addition, the stereo-enantiomer of Peptide 69, Peptide 69D (rrwrivvirvrr-CONH_2_), and an ornithine exchange variant Peptide 69L-R (OOWOIVVIOVOO-CONH_2_) were synthesized and tested using MAP (see Figure 6). In the first experiments at 20 µg/mL, we noticed that some D-peptides showed toxicity (reduced growth) against Mycobacteria (for TB see Section 3.4) at higher concentrations. In consequence, this study was performed at 10 µg/mL instead of 20 µg/mL. Except for Peptide 14L-KR, the growth-enhancing activity remained, with Peptide 14D and 14D-rev even showing some small improvement in activity, especially the longer the experiment lasted. This indicated that the L-peptide might be inactivated by proteases whereas the D-peptide continues to act, with a difference seen from day 15 onwards.

**Figure 7:**
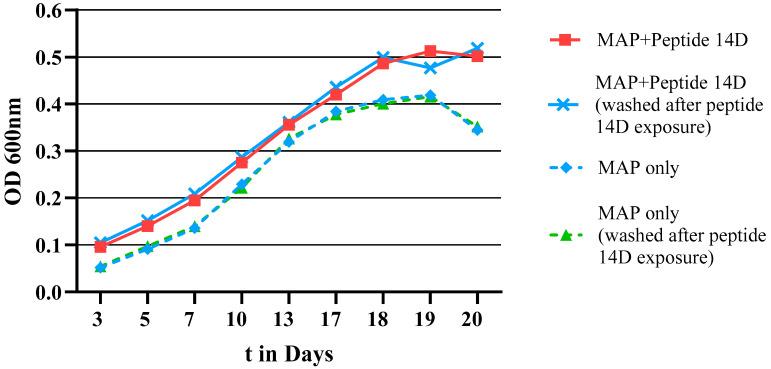
Growth curve of MAP monitored via the absorbance at 600 nm over 20 days. The first experiment compared MAP without treatment (MAP only) and treatment with 20 µg/mL of Peptide 14D (MAP + Peptide 14D). After 20 days, the MAP + Peptide 14D-treated sample was intensively washed, diluted in new media, divided, and growth-monitored. One part was treated again with Peptide 14 at 20 µg/mL (MAP + Peptide 14D washed after Peptide 14D exposure), whereas the other was without treatment (MAP only washed after Peptide 14D exposure).

Peptide 14D was then used to test if growth enhancement was retained after the peptide was removed. MAP was grown with and without Peptide 14D at 20 µg/mL and the growth was monitored by absorbance measurement at 600 nm (see Figure 7). After 20 days, the Peptide 14D-treated sample was intensively washed, diluted, and split into two samples, with one exposed for a second time to Peptide 14D at 20 µg/mL, and the other grown in Peptide 14D-free media. Washed samples reverted to growth curves similar to the control, showing that growth stimulation by Peptide 14D was reversible.

### 3.4. Peptides Tested on Mycobacterium Tuberculosis

With an estimated 10.6 million TB cases in 2021 causing 1.6 million TB-related deaths worldwide [3] it is undoubtfully clear that new diagnostics and treatments are of utmost importance. Therefore, the next question to answer was if those peptides can also affect the MTB growth and, if so, can they improve recovery and diagnostic culture confirmation of MTB? Because of the slow growth of this organism, the time to positivity (TTP) from clinical samples is very high compared to many other bacteria. For example, one study reported an average TPP of 14 days using the Mycobacterium Growth Indicator Tube (MGIT) system (Becton Dickinson, Allschwil, Switzerland), with all positive samples detected within 28 days [38]. We aimed to identify peptides that could reduce the TTP in a commercial MTB test. In consequence, the first screen was performed by using the MGIT system.

A subset of peptides previously tested against *Mycobacterium avium* subsp. *paratuberculosis* (MAP) was also assessed for their impact on *Mycobacterium tuberculosis* (MTB). The evaluation was carried out using the BD BACTEC MGIT320 system, employing the MGIT (Mycobacteria Growth Indicator Tube) to measure the fluorescence of a specific dye as growth units over time. This assay relies on the depletion of oxygen due to bacterial growth, which activates the fluorescence signal.

During preliminary experiments, Peptide 14 exhibited a hormetic response in MTB across a wide range of concentrations. Considering our earlier observations of growth inhibition in MTB using some of the peptides from this study [10], we decided to adjust the concentration for all subsequent investigations, reducing it from 20 µg/mL to 10 µg/mL.

**Figure 8:**
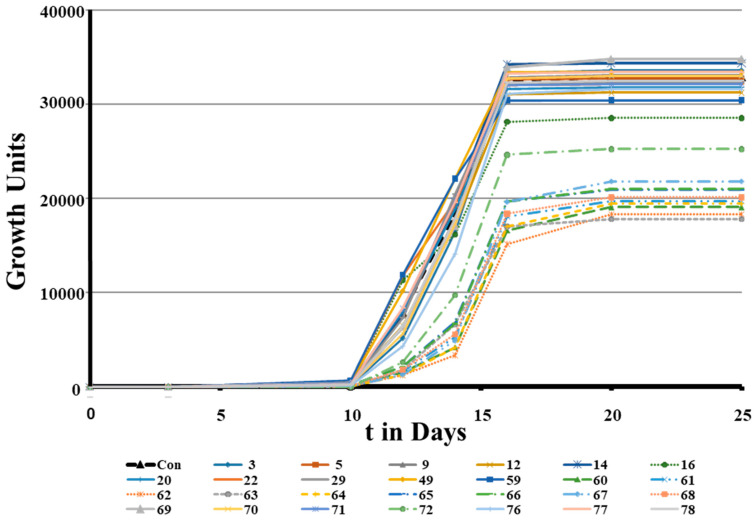
Growth curves of *M. tuberculosis* in the presence of different peptides at 10 µg/mL determined using MGIT in a BD BACTECMGIT 320 system. The numbers are the peptide identifications used in Table S1.

A similar activity pattern was observed with MTB as had been with MAP, see Figure 8. Out of 27 peptides screened at 10 µg/mL, two peptides showed slight improvements (Peptide 69 and Peptide 14), 12 showed similar activity to the control, and 13 peptides showed less growth than the control.

**Figure 9:**
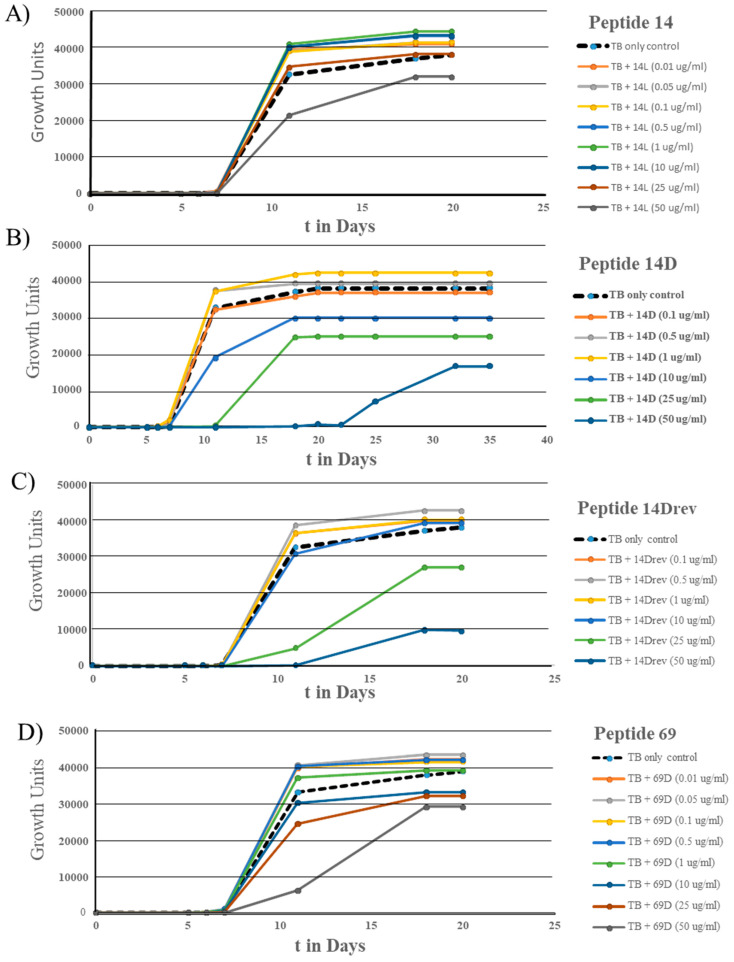
Growth curves of *M. tuberculosis* in the presence of different peptides: (**A**) Peptide 14, (**B**) Peptide 14D, (**C**) Peptide 14Drev, and (**D**) Peptide 69D at a range of concentrations, determined using MGIT in a BD BACTECMGIT 320 system.

In further experiments, dose dependency was measured for a selected set of peptides (see Figure 9). The experiments included four peptides: Peptides 14, 14D, 14Drev, and 69D. Peptide 14 displayed a hormetic response across a broad concentration range (10 ng to 10 µg/mL), with growth inhibition observed only at 50 µg/mL. Peptide 14D exhibited a hormetic response at two concentrations, 0.5 and 1 µg/mL, but higher concentrations (10 µg/mL and above) resulted in growth inhibition. Peptide 14Drev, which shares the same amino acids as Peptide 14D, showed slightly lower toxicity, displaying a hormetic response between 0.1 and 1 µg/mL, with inhibitory effects starting at 25 µg/mL. On the other hand, Peptide 69D demonstrated a hormetic response within the concentration range of 10 ng/mL to 500 ng/mL, with some early activity noted at 1 µg/mL. Growth inhibition for Peptide 69D was observed starting from 10 µg/mL. In summary, all three D-enantiomers were found to be more toxic towards *M. tuberculosis* compared to Peptide 14, but at lower concentrations (1 µg/mL for Peptide 14D, 0.5 µg/mL for Peptide 14Drev, and 10 ng/mL for Peptide 69D), a strong sustained growth stimulation was observed. Peptide 14 showed the widest concentration range with growth stimulation (10 ng/mL to 10 µg/mL).

**Figure 10:**
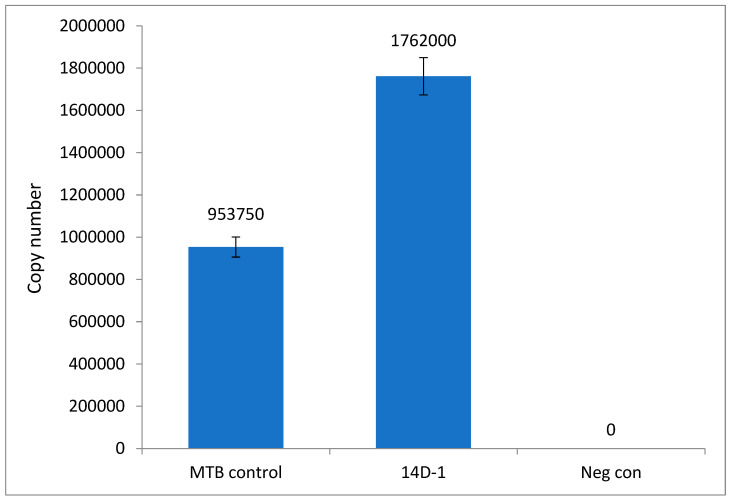
qPCR analysis of *M. tuberculosis* in the absence (MTB control) and presence of 14D at 1 µg/mL.

To further confirm the effect of 14D on *M. tuberculosis*, a quantitative analysis of copy number was performed, see Figure 10. As DNA was extracted when *M. tuberculosis* control went positive, it gave further insight into the effect of the peptide on *M. tuberculosis* growth in comparison to the control. This showed that *M. tuberculosis* spiked with 14D at 1 µg/mL not only had gone positive on the MGIT system before the control but also had a higher copy number. This correlates with our hypothesis that 14D at this concentration accelerates growth. 

**Figure 11:**
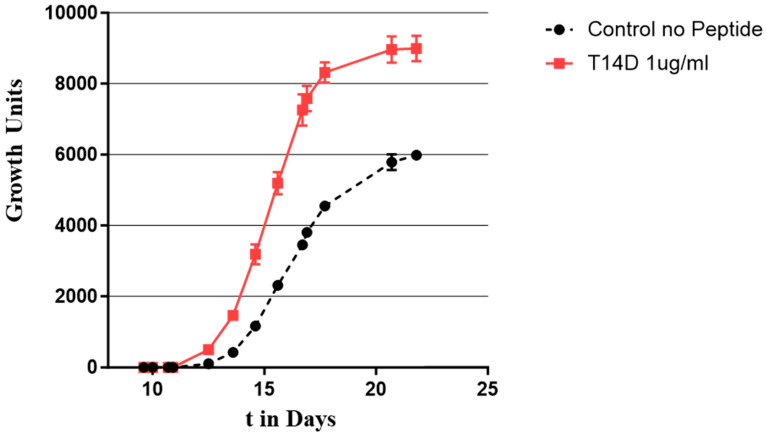
Growth curve of low-inoculum multidrug-resistant (MDR) *Mycobacterium tuberculosis* treated with Peptide 14D at 1 µg/mL showing increased growth (red diamonds) compared with untreated control (black circles).

Whilst culturing of MTB is a very slow method, it is currently the only one to allow the determination of any drug resistance. Drug resistance is becoming a problem in the effective treatment of TB; therefore, culturing MTB remains essential. It was thus important to check if the peptide also affects multidrug-resistant MTB. The effect of Peptide 14D at 1 µg/mL was then tested on a multidrug-resistant strain of *M. tuberculosis* (see Figure 11). Indeed, Peptide 14D could affect the growth of multidrug-resistant MTB, especially by reducing the lag time before growth starts. In addition, the change in the slope of the growth curves indicated a faster division rate.

After those encouraging results, the next set of experiments was designed to show whether peptides from our library (most of them designed) and naturally occurring peptides could reduce the time to positivity (TTP) using standard automated MGIT using a BD BACTECMGIT 320 system widely used for clinical testing (see Table 1). That would open up the possibility to develop them into enhancers of MTB diagnostics by culture. We focused on peptides with high activity at low concentrations, which would be best for development as an adjunct for currently used commercial products, since most TB tests are performed in low- and middle-income countries and the cost of goods is critical. Therefore, the screens were performed at 10 µg/mL and a technical repeat was introduced in the case that observed differences were very small. Since the effect of the peptides depends on the inoculum, two inocula sizes were used, resulting in TTP values of 7 and 15 days for the untreated control. A total of 13 (59, 69, 61, 12, 71, 14, 5, 16, 20, 60, 49, 9, and 78) out of 25 tested peptides showed an overall average larger than 5% decrease in TTP (see Table 1). As observed before, small changes in the sequence led to strong effects; for example, peptides 59–61 showed strong effects, whereas very similar peptides (62 and 63) delayed TTP, especially in low inocula. Similar to the growth curves presented in Figure 1 when using MAP, peptides 69, 12, and 14 induced a strong hormetic response also in MTB (see Table 1). Peptide 69 showed the 2nd strongest response, 12 the 4th, and 14 the 6th. Notably, the human cathelicidin LL37 showed a lower TTP as well. Growth curves monitor the complete growth of the microorganism, while TTP only relies on the early phase of growth, and this is the most important parameter with regard to diagnostics. As demonstrated above, Peptide 14D showed similar activity to Peptide 14 but at lower concentrations. Since for an application in routine MTB diagnostics low costs of goods are important, a short peptide used at low concentration would be ideal. Peptide 14 was ranked as the sixth most active, but second for the short 9mer peptides tested. Peptide 14D was therefore selected to test the concentration effect on the TTP in MGIT using a BD BACTECMGIT 320 system (see Table 2). These inocula without peptide treatment resulted in a TTP of 6 days. The addition of peptides showed faster TTP values within a concentration range between 0.1 and 1 µg/mL, whereas 1 µg/mL had the strongest effects, with an average increase of TTP of 21.6%. The reduction in time until positivity was between 17 and 38 h. At 10 µg/mL, the time to positivity was increased by 8 and 20 h, respectively. At 50 µg/mL, a strong delay of growth was observed, taking about triple the number of days to reach positivity. The data correlate well with the growth observed over 20 days (see Figure 9). In summary, Peptide 14D at a concentration of 1 µg/mL showed the strongest effect on the TTP measure so far. In future research, other peptides that outperformed Peptide 14 could be tested in their D-enantiomer forms in a concentration-dependent manner to find even more active peptides.

Peptide 14D was used to check if other machines that are routinely used in medical microbiology for the detection of mycobacteria show shorter TTP as well. BacT/Alert (Biomerieux, Marcy-l’Étoile, France and VersaTrek (ThermoFisher, Waltham, MA, United States) were tested, and shorter TTP was shown for both systems (see Figure S1 in the Supplementary Materials).

### 3.5. Testing Naturally Occurring Antimicrobial Peptides against M. tuberculosis

A screen was performed with naturally occurring peptides (*n* = 43, crude peptides), synthetic fragments of naturally occurring peptides (*n* = 11, crude) and short-designed peptides (*n* = 5, crude) taken from the ADP3 (http://aps.unmc.edu/AP/) (accessed on 29 August 2023) and confirmed for their antimicrobial activity against methicillin-resistant *Staphylococcus aureus* (MRSA) (unpublished results). In two independent experiments, their effect on TTP of *M. tuberculosis* at 10 µg/mL was determined, inoculated from an exponentially grown stock culture and emulsified before inoculation by passing through a G25 needle (see Table 3). Taking into consideration experiment 1 and experiment 2 (see Table 3), 44 peptides showed an average faster TTP larger than 5%. Nine peptides showed a faster TTP of 10% or more. All of these peptides showed a consistent effect between the two screens; however, the protegrin-1 fragment, where the last two amino acids are omitted, showed a very strong effect only in the second screen. Full-length Protegrin-1 shows antibacterial as well as anticancer activity [39,40]. Lasioglossin LL-III, the peptide inducing the second-best hormetic response, was isolated from the eusocial bee *Lasioglossum laticeps* and can kill Gram-positive and Gram-negative bacteria and various cancer cells; however, it is only mildly toxic against human cells. An internal target was suggested and it was shown that the peptide does not disrupt the membrane (nonlytic) [41,42]. The third-best peptide in the table was the full-length peptide Ranacyclin E from the frog skin of *Rana esculenta* [43]. It is active against Gram-positive bacteria, some Gram-negative, like *Yersinia pseudotuberculosis*, and some fungi. The fourth most active peptide is GF-17. It is a fragment of the human antimicrobial peptide LL37, a cathelicidin. This fragment was described with strong antibacterial and antiviral activity [44,45]. The full-length peptide LL37 showed a similar but less pronounced effect (see Table 1, Peptide 78).

## 4. Discussion

Antimicrobial peptides are naturally occurring molecules in microbes, plants, and animals that perform a variety of different biological functions, including antimicrobial and immunomodulatory activities. By screening short artificial peptides for their antibacterial activity against the very slow fastidious pathogen *Mycobacterium avium* subspecies *paratuberculosis* (MAP), we discovered 13 peptides that had growth-stimulating activity, with Peptide 14 and Peptide 69 among the peptides showing the strongest effects (see Table S1 and Figure 1). A comparison of peptide sequences revealed that small changes in the sequence could induce dramatically different effects on growth. For example, the peptides in Figure 3 show three 12mer peptides, with the conserved core motif RIVVIR found in the bovine AMP bactenecin. Peptide 70 (RLARIVVIRVRR-CONH_2_) has no activity on the growth of MAP. Changes of only two amino acids at the N-terminal region increased positive charge (R) and increased hydrophobicity as well as the ability to integrate into membranes (W) consequently showing an acceleration of growth of MAP, Peptide 69 (RRWRIVVIRVRR-CONH_2_). Reducing the positive charge (A) and increasing hydrophobicity and ability to interact with membranes (A, W) at the C-terminal region showed a reduction of growth in MAP, Peptide 72 (RLARIVVIRWAR-CONH_2_). Further studies revealed that Peptide 14 displayed growth stimulation in both slow- and some selected fast-growing mycobacterial species. Growth stimulation was strongest in low-load inocula and was reversible (Figure 5 and Figure 7). Modifications of peptides 14 and 69 showed that the stereoisomer Peptide 14D, Peptide 14Drev, and Peptide 69D (all containing only D-amino acids) further increased growth stimulation of MAP at 10 µg/mL, indicating that interactions were negated by stereoselectivity but, rather, improved (Figure 6). Furthermore, studies of such D-enantiomers on MTB showed a reduced concentration range compared to all the L-peptides (see below).

Further studies using MTB identified the same two peptides (14 and 69) that improved growth. Dose dependency was measured for four peptides (14, 14D, 14Drev, and 69D). Overall, the peptides’ effects on cell growth were dose-dependent, showing hormetic response in a dose-dependent manner. At low concentrations, the peptides had stimulatory effects on cell growth, while at higher concentrations, they led to growth inhibition. Peptide 69D exhibited the least growth inhibition among the D-enantiomers tested. The dose dependency of Peptide 14 showed the largest range of activity (50 ng/mL and 10 µg/mL) of the growth stimulatory effect for all peptides tested. All tested D-peptides showed a smaller activity range for growth stimulatory effect and, in addition, revealed a stronger antimicrobial effect at higher concentrations (see Figure 8 and Table 3). Conducting dose-dependency studies on a diverse range of peptides in further research could enhance our understanding of the optimal peptide concentrations to be used in subsequent screenings. This approach may lead to the identification of a broader set of peptides that exhibit a hormetic response against slow-growing Mycobacteria. Peptide 14D stimulated the growth of both antibiotic-sensitive and multidrug-resistant *M. tuberculosis* similarly.

Nine artificial peptides were identified that consistently lowered TTP for *M. tuberculosis* using culture techniques and cut-offs in line with routine diagnostic protocols. In addition, we identified 44 peptides from the database ADP3 that lowered TTP for MTB growth by 5–15.3% and 9 peptides which lowered TTP by 10% or more (Table 1 and Table 2).

**Figure 12:**
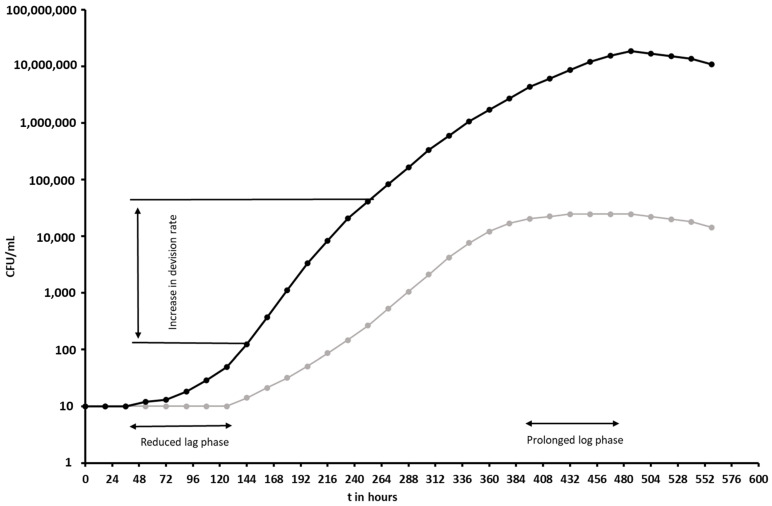
Modelled growth curves of *Mycobacterium tuberculosis,* untreated in grey and treated with a peptide with a hormetic effect in black.

We observed that peptides are active as low as 10 ng/mL; we thus hypothesise that these types of peptides are sensed by mycobacteria and, in slow-growing mycobacteria in particular, induce a hormetic response. It is well described that Gram-positive and Gram-negative bacteria can sense antimicrobial peptides, for example, PhoP/PhoQ, a two-component sensor/regulator system in *Salmonella typhimurium* and Aps in *Staphylococcus epidermidis* [46]. We observed three changes in the growth of the mycobacteria tested. The first change is the faster transition from the lag phase into the log phase compared to untreated cells. Lower inoculation sizes tend to have a longer lag phase and therefore the effects of the peptide are most pronounced at lower inoculum sizes. The second change observed is a faster decision rate, which was more pronounced with MAP and not consistently seen with *Mycobacterium tuberculosis*. Further research is needed to investigate this in more detail. These two changes give hope that those peptides can indeed be used to improve mycobacterial diagnostics by culture. The third change observed was a longer continuation of the log phase compared to the untreated control, indicating higher cell numbers at the end of the growth phase. These could be interesting for research purposes. In Figure 12, all three changes are presented in two modelled growth curves. Further results for the mode of action will be published in a separate paper.

The WHO estimates that about 3–4 million culture tests are performed worldwide to grow Mycobacteria, especially *M. tuberculosis*. Phenotypic antibiograms require culture and provide the most accurate way to determine resistance and inform on management therapy. Our discovery that the strongest effect of these peptides occurs at very low bacterial loads suggests that the sensitivity of culturing MTB could be improved. In consequence, this discovery represents a platform for the product development of a growth enhancer for different mycobacteria. These products have been introduced to the market. A clinical study evaluating growth-enhancing peptides in combination with a new sample preparation procedure has been performed and showed higher sensitivity (publication will be submitted separately). Further work is needed to deduce the full mechanism of action of these agents and their possible role in the recovery of latent, viable unculturable forms of pathogenic mycobacteria.

We conclude that some natural and artificial cationic antimicrobial peptides demonstrate a hormetic effect on mycobacteria, mainly on slow-growing mycobacteria. Whereas a range of different sequences showed activity, small changes in the sequence can destroy the activity. Inverse and retro-inverse peptides also displayed activity, but at a smaller concentration range and displaying stronger toxicity. The hormetic effect we observed is dose-dependent and occurred at a concentration as low as 10 ng/mL. In addition, the effect is dependent on the starting inocula; the lower the inocula, the greater the effect.

## Figures and Tables

**Table 1:** Effect of selected peptides at 10 µg/mL on the time to positivity (TTP) of *M. mycobacterium* measured in MGIT in a BD BACTECMGIT 320 system. Experiments 1a and 1b are technical repeats, independent of experiments 2a and 2b (technical repeats). Since the effect of the peptides depends on the inoculum, two inocula sizes were used, one less diluted, resulting in a TTP of 7 days, and a diluted one, with an TTP of 15 days. The averages for % faster than control were calculated for experiments 1a and 1b as well as 2a and 2b. The overall average was then calculated from these two average values.

Peptide Code	Sequence	Time to Positivity (Days.Hours) Experiment 1a	Time to Positivity (Days.Hours) Experiment 1b	Time to Positivity (Days.Hours) Experiment 2a	Time to Positivity (Days.Hours) Experiment 2b	Average % Faster than Control
**TB control (no peptide)**		7.02	7.01	15.01	15.20	
**59**	**ILKWKRKWWKWRR**	5.17	5.22	13.19	12.22	14.5
**69**	**RRWRIVVIRVRR**	6.02	6.02	13.12	13.02	12.8
**61**	**ILKWKTKWWKWFR**	6.20	5.23	13.11	14.21	10.4
**12**	**RRRIKIRWY**	6.14	5.20	14.08	14.22	10.2
**71**	**RLWRIVVIRVKR**	6.04	6.06	14.01	14.02	9.4
**14**	**WKIVFWWRR**	6.01	6.12	13.23	14.01	9.1
**5**	**FFIYVWRRR**	5.21	6.08	14.06	14.16	8.8
**16**	**RLKRWWKFL**	6.01	5.22	14.17	14.20	8.6
**20**	**KWKWWWRKI**	6.11	6.00	14.01	14.16	8.3
**60**	**ILKWKIFKWKWFR**	6.03	6.02	14.19	14.16	7.9
**49**	**VRLRIRVRVIRK**	5.22	6.06	15.12	15.02	6.2
**9**	**KRRWRIWLV**	6.07	6.04	15.08	14.21	5.8
**78**	**LLGDFFRKSKEKIGKEFKRIVQRIKDFLRNLVPRTES**	6.08	6.09	14.15	15.00	5.8
**77**	**VRLRIRVAVIRA**	6.14	6.04	14.22	15.03	5.0
**29**	**RWRWWWRVY**	6.13	6.00	15.12	15.01	4.9
**22**	**IRMRIRVLL**	6.00	6.07	15.19	15.10	4.7
**70**	**RLARIVVIRVRR**	6.03	6.19	14.22	15.06	4.2
**3**	**WKWRVRVTI**	6.08	6.17	14.22	15.02	4.0
**68**	**RLRRIVVIRVAR**	6.09	6.17	15.00	15.23	2.3
**65**	**RGARIVVIRVAR**	6.16	6.03	16.11	16.01	0.8
**72**	**RLARIVVIRWAR**	6.22	7.01	15.01	15.13	−0.2
**67**	**RLWRIVVIRVAR**	6.19	6.11	16.00	16.05	−0.4
**64**	**RLARIVVIRVAR**	6.19	6.16	15.21	16.13	−1.4
**76**	**VRWRIRVAVIRA**	6.13	6.16	16.08	16.14	−1.4
**62**	**ILKWKMFKWKWFR**	6.07	7.02	16.20	17.16	−3.4
**66**	**RRARIVVIRVAR**	6.05	6.16	17.06	17.16	−3.5
**63**	**ILPWKWPWWPWRR**	6.22	6.22	17.01	17.10	−5.5

**Table 2:** Effect of Peptide 14D at different concentrations on the time to positivity (TTP) of *M. tuberculosis* in two independent experiments measured in MGIT in a BD BACTEC MGIT 320 system.

Peptide 14D Concentration (µg/mL)	Time to Positivity (Days.Hours) Experiment 1	Time to Positivity (Days.Hours) Experiment 2	Average % Faster than Control
**0**	6.19	6.12	
**0.1**	5.12	5.19	15.0
**0.5**	5.17	5.17	14.1
**1**	5.05	5.08	21.6
**10**	7.03	7.08	−7.3
**25**	9.21	9.17	−48.7
**50**	19.15	21.5	−207.6

**Table 3:** A screen of 43 naturally occurring peptides, 11 fragments of naturally occurring peptides, and 5 designed peptides, all taken from the APD3, was performed at 10 µg/mL in two independent experiments on the TTP of *M. tuberculosis* measured in MGIT in a BD BACTECMGIT 320 system. Fragments are labelled with the * symbol.

Name/Source	Sequence	Time to Positivity (Day.Hours) Experiment 1	Time to Positivity (Day.Hours) Experiment 2	Average % Faster than Control
**Control (TB only)**		**11.10**	**9.10**	
Protegrin 1/Pig *	**RGGRLCYCRRRFCVCV**	**10.23**	**6.22**	**15.3**
**Lasioglossin LL-III/Bee**	**VNWKKILGKIIKVVK**	**9.18**	**8.08**	**13.1**
**Ranacyclin E/Frog**	**SAPRGCWTKSYPPKPCK**	**10.07**	**8.01**	**12.2**
GF-17/Human *	**GFKRIVQRIKDFLRNLV**	**10.02**	**8.07**	**11.8**
Arenicin 1/Annelid worm *	**RWCVYAYVRVRGVLVR**	**10.15**	**8.02**	**10.5**
PP13/Wasp *	**IRLHRLYTWKATIYTR**	**10.17**	**8.02**	**10.2**
**Peptide B9/Frog**	**FLPLIAGLIGKLF**	**10.19**	**8.01**	**10.0**
**CM15/Bee venom**	**KWKLFKKIGAVLKVL**	**10.19**	**8.01**	**10.0**
**Macropin 1/Wasp**	**GFGMALKLLKKVL**	**10.12**	**8.07**	**10.0**
Salusin-beta/Scorpion *	**FIFIRWLLKLGHHGRA**	**10.18**	**8.03**	**9.8**
GLK-19/Designed *	**KKLLGKLLKKLGKLLL**	**10.17**	**8.04**	**9.7**
Odorranain-H1/Frog *	**FGKILGVGKKVLCGLS**	**10.19**	**8.03**	**9.6**
**Temporin-HN1/Frog**	**AILTTLANWARKFL**	**10.23**	**8.01**	**9.3**
**CPF-AM4/Frog**	**GLGSLVGNALRIGAKLL**	**10.17**	**8.06**	**9.3**
**Tachyplasin II/Crab**	**RWCFRVCYRGICYRKCR**	**10.18**	**8.06**	**9.1**
**Mastoparan-VT6/Wasp**	**INLKAIAALVKKLL**	**10.21**	**8.04**	**9.0**
**Temporin-1TSa/Frog**	**FLGALAKIISGIF**	**11.00**	**8.02**	**8.9**
**Temporin-1DRa/Frog**	**HFLGTLVNLAKKIL**	**10.18**	**8.09**	**8.5**
**Temporin-HN2/Frog**	**NILNTIINLAKKIL**	**11.03**	**8.02**	**8.4**
NRC-15/Fish *	**WGKLFKLGLHGIGLLH**	**10.21**	**8.07**	**8.3**
**CM-3/Designed**	**ALKAALLAILKIVRVI**	**11.04**	**8.03**	**8.0**
Piscidin 1/Fish *	**FFHHIFRGIVHVGKTI**	**10.18**	**8.12**	**7.8**
**Eumenitin/Wasp**	**LNLKGIFKKVASLLT**	**11.00**	**8.08**	**7.6**
**OdVP2/Wasp**	**ILGIITSLLKSLGKK**	**10.22**	**8.11**	**7.3**
**Clavanin C/Sea squirt**	**VFHLLGKIIHHVGNFV**	**10.21**	**8.12**	**7.2**
**P-04/Tunicate**	**FFPYAALKWLRKLLKK**	**11.02**	**8.08**	**7.2**
**Temporin L/Frog**	**FVQWFSKFLGRIL**	**11.06**	**8.05**	**7.1**
Protegrin 4/Pig *	**GRLCYCRGWICFCVGR**	**11.11**	**8.01**	**7.1**
D28/Designed *	**FLGVVFKLASKVFPAV**	**11.02**	**8.09**	**7.0**
**TsAP-1/Scorpion**	**FLSLIPSLVGGSISAFK**	**11.07**	**8.05**	**7.0**
**Brevinin-1Ta/Frog**	**FITLLLRKFICSITKKC**	**11.07**	**8.05**	**7.0**
**Bombolitin V/Bee**	**INVLGILGLLGKALSHL**	**10.22**	**8.13**	**6.8**
**Temporin-LTb/Frog**	**FIITGLVRGLTKLF**	**11.07**	**8.06**	**6.7**
**Temporin-1Cb/Frog**	**FLPLFASLIGKLL**	**11.08**	**8.06**	**6.6**
**Hylin a1/Frog**	**GAILPLALGALKNLIK**	**11.14**	**8.02**	**6.3**
**Arenicin-2/Lugworm**	**YAYVRIRGVLVRYRRC**	**11.13**	**8.03**	**6.3**
**Temporin-PTa/Frog**	**FFGSVLKLIPKIL**	**11.07**	**8.08**	**6.3**
**Decoralin/Wasp**	**SLLSLIRKLIT**	**11.12**	**8.04**	**6.3**
**Pd_mastoparan PDD-B/Wasp**	**INWLKLGKKILGAL**	**11.06**	**8.09**	**6.3**
**NRC-15/Flounder**	**GFWGKLFKLGLHGIGL**	**11.02**	**8.14**	**5.9**
**Clavanin E/Sea squirt**	**LFKLLGKIIHHVGNFV**	**11.05**	**8.12**	**5.8**
**Temporin 1Vb/Frog**	**FLSIIAKVLGSLF**	**11.10**	**8.08**	**5.8**
**Alyteserin-2b/Toad**	**ILGAILPLVSGLLSNKL**	**11.18**	**8.02**	**5.6**
Polyphemusin/Crab *	**WCFRVCYKGFCYRKCR**	**11.05**	**8.13**	**5.6**
**IsCT2/Scorpion**	**IFGAIWNGIKSLF**	**10.23**	**8.21**	**4.9**
**Indolicidin/Cow**	**ILPWKWPWWPWRR**	**11.03**	**8.18**	**4.8**
**Temporin-SN1/Frog**	**FFPFLLGALGSLLPKIF**	**10.22**	**8.23**	**4.6**
**Pelophylaxin-4/Frog**	**ILPFLAGLFSKIL**	**11.05**	**8.18**	**4.5**
**P9/Designed**	**KRRWRIWLV**	**11.10**	**8.14**	**4.4**
**Brevinin 1Tb/Frog**	**LVPLFLSKLICFITKKC**	**11.10**	**8.15**	**4.2**
**Casecidin 17/Cow**	**YQEPVLGPVRGPFPIIV**	**11.12**	**8.14**	**4.1**
**Brevinin-1DYc/Frog**	**LLAGLPKLLCLFFKKC**	**11.10**	**8.16**	**4.0**
**Temporin-1Oc/Frog**	**FLPLLASLFSRLF**	**11.10**	**8.17**	**3.8**
**Temporin A/Frog**	**FLPLIGRVLSGIL**	**11.02**	**9.00**	**3.7**
Fallaxidin/Frog *	**LSFLPKVIGVIGHLIH**	**11.18**	**8.13**	**3.2**
**Pantinin-3/Scorpion**	**FLSTIWNGIKSLL**	**11.08**	**8.23**	**2.8**
**Temporin-1Vc/Frog**	**FLPLVTMLLGKLF**	**11.12**	**8.21**	**2.5**
P8/Designed *	**FFIYVWRRR**	**11.16**	**8.22**	**1.6**
**Tritrpticin/Pig**	**VRRFPWWWPFLRR**	**11.08**	**9.13**	**−0.3**

## Data Availability

The vast majority of data is presented in the manuscript. Further data presented in this study are available on request from the corresponding author.

## Supplementary Materials

The following supporting information can be downloaded at: https://www.mdpi.com/article/10.3390/microorganisms11092225/s1. Table S1: Screening of 75 peptides against MAP. Figure S1: BacT/Alert and VersaTrek test.
