# Supplementary material for: Discovery of Antimicrobial Peptides That Can Accelerate Culture Diagnostics of Slow-Growing Mycobacteria Including Mycobacterium tuberculosis
Source: Microorganisms. 2023 Sep 2;11(9):2225. doi: 10.3390/microorganisms11092225 (PMC10536189; doi:10.3390/microorganisms11092225)
Supplement: Supplementary file 1 [file microorganisms-11-02225-s001.zip › microorganisms-2515233-supplementary.pdf]

Supplement

Table S1

A

Condition	Day 1	Day 5	Day 8	Day 12	Day 15	Day 19	Day 22
MAP (control)	0.038	0.231	0.330	0.467	0.545	0.633	0.699
MAP + Peptide 1	0.038	0.067	0.251	0.202	0.286	0.374	0.579
MAP + Peptide 2	0.056	0.195	0.244	0.219	0.295	0.384	0.639
MAP + Peptide 3	0.044	0.118	0.224	0.272	0.286	0.408	0.582
MAP + Peptide 4	0.016	0.184	0.257	0.266	0.287	0.333	0.574
MAP + Peptide 5	0.029	0.117	0.191	0.253	0.351	0.438	0.549
MAP + Peptide 6	0.041	0.148	0.174	0.261	0.284	0.447	0.606
MAP + Peptide 7	0.026	0.202	0.246	0.189	0.313	0.453	0.620
MAP + Peptide 8	0.023	0.102	0.232	0.382	0.421	0.501	0.559
MAP + Peptide 9	0.033	0.204	0.352	0.406	0.516	0.550	0.624
MAP + Peptide 10	0.016	0.238	0.330	0.472	0.579	0.626	0.718
MAP + Peptide 11	0.027	0.272	0.327	0.526	0.583	0.627	0.687
MAP + Peptide 12	0.029	0.165	0.349	0.456	0.486	0.547	0.644
MAP + Peptide 13	0.035	0.176	0.335	0.383	0.527	0.574	0.701
MAP + Peptide 14	0.037	0.272	0.411	0.478	0.601	0.651	0.711
MAP + Peptide 15	0.029	0.133	0.095	0.160	0.189	0.287	0.465
MAP + Peptide 16	0.013	0.213	0.271	0.393	0.530	0.542	0.619
MAP + Peptide 17	0.047	0.061	0.105	0.154	0.182	0.284	0.335
MAP + Peptide 18	0.047	0.115	0.238	0.398	0.540	0.633	0.624
MAP + Peptide 19	0.008	0.145	0.383	0.453	0.543	0.610	0.616
MAP + Peptide 20	0.033	0.097	0.187	0.229	0.375	0.559	0.618
MAP + Peptide 21	0.050	0.121	0.149	0.233	0.384	0.453	0.598
MAP + Peptide 22	0.038	0.091	0.160	0.199	0.418	0.421	0.694
MAP + Peptide 23	0.032	0.100	0.146	0.208	0.423	0.454	0.642
MAP + Peptide 27	0.047	0.117	0.159	0.231	0.401	0.489	0.653
MAP + Peptide 28	0.043	0.090	0.249	0.301	0.345	0.434	0.581

B

Condition	Day 1	Day 5	Day 8	Day 12	Day 15	Day 19	Day 22
MAP (control)	0.050	0.217	0.313	0.415	0.451	0.530	0.606
MAP + Peptide 29	0.047	0.146	0.186	0.343	0.381	0.493	0.581
MAP + Peptide 30	0.037	0.194	0.272	0.274	0.461	0.532	0.666
MAP + Peptide 31	0.028	0.122	0.193	0.387	0.368	0.525	0.605
MAP + Peptide 35	0.047	0.127	0.180	0.199	0.414	0.483	0.490
MAP + Peptide 36	0.023	0.137	0.163	0.319	0.394	0.518	0.588
MAP + Peptide 38	0.046	0.176	0.188	0.212	0.308	0.491	0.602
MAP + Peptide 39	0.031	0.156	0.215	0.254	0.363	0.466	0.459
MAP + Peptide 42	0.017	0.165	0.257	0.287	0.317	0.402	0.511
MAP + Peptide 43	0.043	0.136	0.146	0.244	0.453	0.558	0.571
MAP + Peptide 47	0.018	0.110	0.167	0.275	0.387	0.523	0.621
MAP + Peptide 48	0.038	0.154	0.176	0.343	0.458	0.546	0.565
MAP + Peptide 49	0.045	0.157	0.129	0.261	0.380	0.544	0.628
MAP + Peptide 50	0.051	0.113	0.172	0.228	0.308	0.382	0.416
MAP + Peptide 63	0.030	0.140	0.119	0.234	0.394	0.500	0.503
MAP + Peptide 64	0.042	0.134	0.130	0.248	0.474	0.498	0.517
MAP + Peptide 65	0.038	0.139	0.143	0.311	0.422	0.486	0.620
MAP + Peptide 66	0.039	0.107	0.143	0.251	0.328	0.469	0.505
MAP + Peptide 67	0.033	0.222	0.324	0.453	0.560	0.622	0.674
MAP + Peptide 68	0.046	0.125	0.121	0.173	0.403	0.436	0.584
MAP + Peptide 69	0.038	0.246	0.330	0.467	0.614	0.673	0.706
MAP + Peptide 70	0.038	0.125	0.127	0.307	0.393	0.434	0.526
MAP + Peptide 71	0.046	0.145	0.149	0.320	0.458	0.425	0.530
MAP + Peptide 72	0.030	0.098	0.190	0.217	0.264	0.367	0.397
MAP + Peptide 76	0.021	0.193	0.286	0.437	0.532	0.541	0.646
MAP + Peptide 77	0.024	0.193	0.307	0.401	0.451	0.475	0.534

C

Condition	Day 1	Day 5	Day 8	Day 12	Day 15	Day 19	Day 22
MAP (control)	0.052	0.228	0.345	0.438	0.516	0.557	0.631
MAP + Peptide 78	0.055	0.198	0.268	0.381	0.427	0.480	0.549
MAP + Peptide 82	0.036	0.094	0.101	0.128	0.131	0.146	0.147
MAP + Peptide 83	0.042	0.135	0.174	0.205	0.194	0.212	0.209
MAP + Peptide 84	0.045	0.151	0.199	0.240	0.391	0.514	0.532
MAP + Peptide 86	0.037	0.186	0.314	0.451	0.489	0.568	0.597
MAP + Peptide 87	0.030	0.075	0.121	0.151	0.212	0.314	0.331
MAP + Peptide 88	0.062	0.105	0.148	0.177	0.242	0.311	0.415
MAP + Peptide 90	0.029	0.162	0.248	0.376	0.482	0.591	0.594
MAP + Peptide 91	0.059	0.197	0.292	0.400	0.490	0.525	0.598
MAP + Peptide 92	0.037	0.189	0.313	0.368	0.477	0.533	0.573
MAP + Peptide 97	0.039	0.213	0.323	0.376	0.428	0.524	0.578
MAP + Peptide 98	0.051	0.164	0.215	0.275	0.379	0.454	0.500
MAP + Peptide 99	0.043	0.229	0.346	0.440	0.490	0.531	0.625
MAP + Peptide 100	0.040	0.273	0.374	0.417	0.477	0.526	0.623
MAP + Peptide 102	0.038	0.248	0.372	0.467	0.530	0.591	0.687
MAP + Peptide 103	0.036	0.298	0.349	0.471	0.512	0.580	0.692
MAP + Peptide 108	0.031	0.058	0.094	0.196	0.230	0.341	0.351
MAP + Peptide 109	0.031	0.218	0.336	0.444	0.491	0.555	0.637
MAP + Peptide 110	0.038	0.081	0.140	0.167	0.260	0.358	0.412
MAP + Peptide 111	0.032	0.261	0.390	0.440	0.510	0.596	0.625
MAP + Peptide 112	0.043	0.227	0.323	0.425	0.501	0.542	0.670
MAP + Peptide 114	0.037	0.083	0.161	0.181	0.205	0.291	0.387
MAP + Peptide 115	0.032	0.199	0.295	0.380	0.468	0.571	0.633
MAP + Peptide 116	0.033	0.211	0.318	0.381	0.503	0.539	0.613
MAP + Peptide 117	0.043	0.247	0.311	0.386	0.480	0.539	0.676

Table S1: Screening of 75 peptides against MAP, done in 3 sets (A-C). Growth of MAP was monitored by measuring the absorbance at 600 nm. Values are average of two technical repeats.

Figure S1

(A)

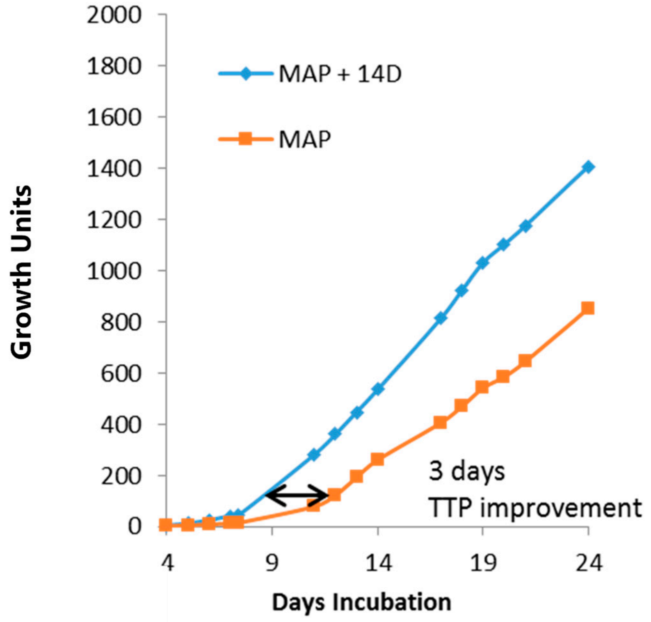

(B)

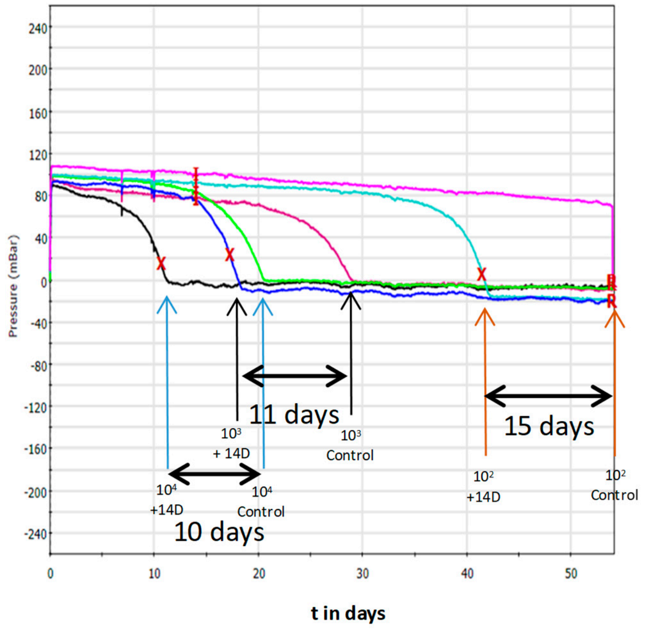

Figure S1: Peptide 14D at 1 µg/mL was used to check if other machines that are routinely used in medical microbiology for the detection of mycobacteria show lower TTP as well. (A) BacT/Alert (Biomerieux, France) was used to detect MAP and (B) VersaTrek (ThermoFisher, US) were tested to detect MTB at different inocula, 10^2, 10^3 and 10^4 . Reduction of TTP in regards to the control is given in days.
